# Effective Surface Nano-Crystallization of Ni_2_FeCoMo_0.5_V_0.2_ Medium Entropy Alloy by Rotationally Accelerated Shot Peening (RASP)

**DOI:** 10.3390/e22101074

**Published:** 2020-09-24

**Authors:** Ningning Liang, Xiang Wang, Yang Cao, Yusheng Li, Yuntian Zhu, Yonghao Zhao

**Affiliations:** 1 School of Materials Science and Engineering, Nanjing University of Science and Technology, Nanjing 210094, China; ningning623@126.com (N.L.); mumuchuntian@gmail.com (X.W.); liyusheng@njust.edu.cn (Y.L.); y.zhu@cityu.edu.hk (Y.Z.); 2 Department of Materials Science and Engineering, City University of Hong Kong, Hong Kong 999077, China

**Keywords:** medium entropy alloy, deformation twinning, dislocation slip, surface nano-crystallization, shot peening

## Abstract

The surface nano-crystallization of Ni_2_FeCoMo_0.5_V_0.2_ medium-entropy alloy was realized by rotationally accelerated shot peening (RASP). The average grain size at the surface layer is ~37 nm, and the nano-grained layer is as thin as ~20 μm. Transmission electron microscopy analysis revealed that deformation twinning and dislocation activities are responsible for the effective grain refinement of the high-entropy alloy. In order to reveal the effectiveness of surface nano-crystallization on the Ni_2_FeCoMo_0.5_V_0.2_ medium-entropy alloy, a common model material, Ni, is used as a reference. Under the same shot peening condition, the surface layer of Ni could only be refined to an average grain size of ~234 nm. An ultrafine grained surface layer is less effective in absorbing strain energy than a nano-grain layer. Thus, grain refinement could be realized at a depth up to 70 μm in the Ni sample.

## 1. Introduction

After decades of fast development in physical metallurgy, dilute alloys and single-principal-element alloys have approached the limit of performance enhancement [1]. However, the trade-off between strength and ductility is still a thorny issue [2]. Different from conventional alloy design, high entropy alloys (HEAs) and medium entropy alloys (MEAs) have attracted immense attention [3,4,5,6,7]. Conventional alloys have configurational entropies, derived from mixing of the alloying components, less than 1R (R = 8.314 J·mol^−1^·K^−1^ is the gas constant); MEAs have configurational entropies in the range between 1R and 1.5R; HEAs have configurational entropies larger than 1.5R [8]. HEAs and MEAs may crystallize into single phase materials due to the configurational entropy maximization effect on solid-solution stabilization. Due to the unique atomic architecture and core effect, HEAs and MEAs exhibit exceptional mechanical properties, including high tensile strength [7,9], high ductility [2,10], excellent fatigue properties [11] and good fracture toughness at cryogenic temperatures [12]. Additionally, some noteworthy physical performances are also obtained for HEAs and MEAs, such as high thermal stability [13], irradiation resistance [14,15], corrosion resistance [8,16] and excellent mechanical behavior [17,18], as well as magnetic properties [19]. Thus, it is believed that both HEAs and MEAs have a huge potential in structural applications, especially for structures servicing in harsh environments.

The deformation mechanisms commonly found in conventional metallic materials, such as dislocation slip and deformation twinning, also play important roles in HEAs and MEAs. However, attributed to the low stacking-fault energy (SFE), short-range ordering effect and local elemental fluctuations, dislocation slip and deformation twinning can be very chaotic in HEAs and MEAs during plastic deformation [6,20,21,22,23,24,25]. It is well known that the propensity for deformation twinning is inversely proportional to the SFE. The SFE of the Cantor HEA is at the lower bond ~20–25 mJ/m^2^ [25]. Thus, the presence of high densities of deformation twinning is found to be a major mechanism of the plastic strain in the Cantor HEA. Deng et al. [24] designed a face-centered cubic (FCC) single-phase Fe_40_Mn_40_Co_10_Cr_10_ HEA that has a large strain-hardening capacity attributed to the high densities of deformation twins and dislocations.

Similar to conventional alloys, single-phase coarse-grained FCC HEAs generally possess high tensile ductility but low yield strength [26,27]. For example, FeMnNiCoCr alloys having average grain sizes of 50 μm and 12 μm show low yield strengths of 95 MPa and 245 MPa, and elongations of 58% and 50%, respectively [27]. Considering the large strain hardening capacities of many high entropy alloys, severe plastic deformation (SPD) seems to be an ideal strategy for grain refinement and strength improvement. In the last 40 years, SPD techniques have been widely used to successfully prepare ultrafine grained (UFG) metals and alloys by means of grain refinement mechanisms [28,29]. According to the Hall–Petch relationship and experimental results, the UFG metals and alloys truly possess high yield strength but unfortunately low tensile ductility. Therefore, breaking the strength–ductility paradox and optimizing the strength–ductility combination are still hot research topics in the SPD field [30,31]. Up to now, many different SPD techniques have been developed, including equal-channel angular pressing (ECAP) [32], high-pressure torsion (HPT) [33], surface mechanical attrition treatment (SMAT) [34] and rotary swaging (RS) [35], etc. SMAT is an effective SPD method for generating a nano-structured surface layer [34,36,37,38,39]. Except for the global strength of SMAT-treated materials being effectively enhanced, wear resistance withstanding common failures on the surface and fatigue properties are also increased significantly [38,39].

Recently, Wu et al. used HPT to process an FeCoCrNi HEA, and significant grain refinement was realized via complicated concurrent nano-band subdivision and high-order hierarchical twinning mechanisms [25]. In addition, a high strain rate deformation may also facilitate grain refinement in medium-entropy alloy [40,41], but the relevant research is limited. Thus, the idea of using the SMAT method to improve the mechanical properties of MEAs has come through our mind. It is worth mentioning that some SMAT methods can break the size constraints of the traditional SPD method with an open specimen chamber, and thus process materials with possibly unlimited sizes. Therefore, an in-depth understanding of the SMAT of HEAs and MEAs is of significant importance to both engineering applications and scientific research. In this work, the same SMAT treatment was conducted on both Ni_2_FeCoMo_0.5_V_0.2_ MEA (configurational entropy of 1.395R) and commercial purity Ni (CP-Ni). The gradient structures formed in the MEA are compared to that of the CP-Ni to reveal the uniqueness of the SMAT process in MEA from a microscopic point view.

## 2. Materials and Methods

Elemental Ni, Co, Fe, V and Mo were used as raw materials, each having a purity greater than 99.5%. The raw materials with the nominal composition of Ni_2_CoFeV_0.5_Mo_0.2_ were alloyed via the arc-melting method under a high purity argon atmosphere. The compositional homogeneity of the alloy was analyzed by atom probe tomography (APT) [42], which revealed that all the alloying elements (Ni, Co, Fe, V, Mo) are homogenously distributed in a cylinder of ∅ 30 nm × 200 nm, indicating a random solid solution MEA without apparent elemental segregation or second phases. The X-ray diffraction (XRD) pattern revealed the simple FCC structure of the Ni_2_CoFeV_0.5_Mo_0.2_ MEA [42]. A CP-Ni was purchased in the market. All sample materials were annealed at 950 °C for 10 h prior to rotationally accelerated shot peening (RASP) [43]. RASP was conducted at room temperature, for 10 min, using GCr15 bearing steel balls with a diameter of 3 mm and a velocity of 60 m/s.

The microstructures of samples before and after RASP were examined by a scanning electron microscope and a transmission electron microscope. Electron backscattering diffraction (EBSD) analysis was conducted using a field emission Carl Zeiss-Auriga SEM equipped with an Oxford Instruments EBSD system. TEM analysis was performed using a FEI T20 TEM operating at 200 kV. Prior to EBSD analysis, the specimens were carefully ground with SiC papers, and then mechanically polished with colloidal silica suspensions. Finally, all specimens were electro-polished (for polishing MEA:CH_3_COOH:HClO_4_ = 9:1, voltage: 50V; for polishing Ni:H_3_PO_4_:H_2_O = 7:1, voltage: 7V). TEM specimens were mechanically polished to 40 μm thick foils and then prepared by a twin-jet polisher (for polishing MEA:C_2_H_5_OH:HClO_4_ = 9:1; for polishing Ni:HNO_3_:CH_3_OH = 1:2) at −25 °C. The thin foil for EBSD and TEM was sectioned from the plane perpendicular to the treatment surface.

## 3. Results

**Figure 1:**
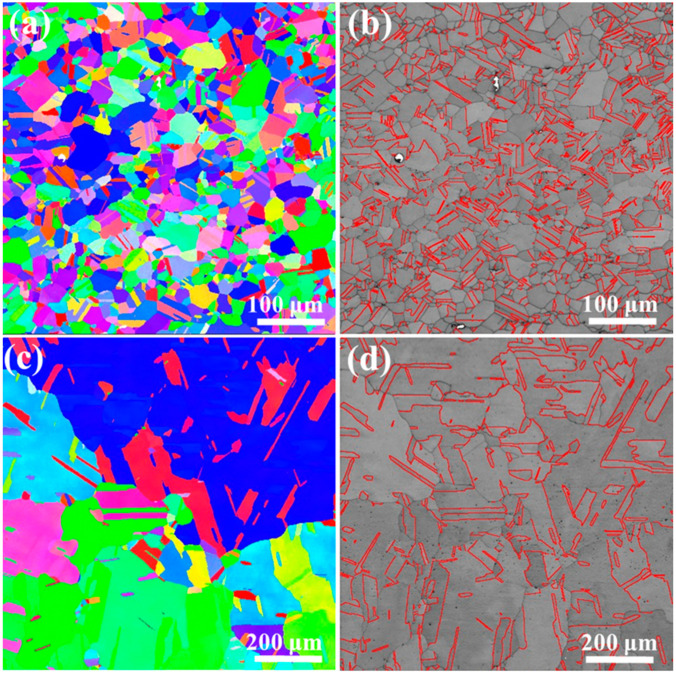
Electron backscattering diffraction (EBSD) maps of annealed sample materials: (**a**) inverse pole figure (IPF) map of Ni_2_FeCoMo_0.5_V_0.2_ MEA, (**b**) twin boundaries in Ni_2_FeCoMo_0.5_V_0.2_ MEA, (**c**) IPF map of CP-Ni, and (**d**) twin boundaries in CP-Ni.

The recrystallized equiaxed microstructures of the annealed FeCoNiMoV MEA are shown in Figure 1a,b. The average grain size of the annealed MEA is ~32 μm. Annealing twins are homogeneously distributed in the grains (Figure 1b). In contrast, the average grain size of the annealed CP-Ni is ~220 μm (Figure 1c), which is much larger than that of the MEA. This is because the diffusion kinetics of the MEA are comparatively low at 950 °C [13]. Annealing twins are also frequently found in the annealed CP-Ni (Figure 1d), but the twin density is clearly lower than that of the MEA (Figure 1b). This is because the CP-Ni possesses a much higher SFE and larger grain sizes than the FeCoNiMoV MEA.

**Figure 2:**
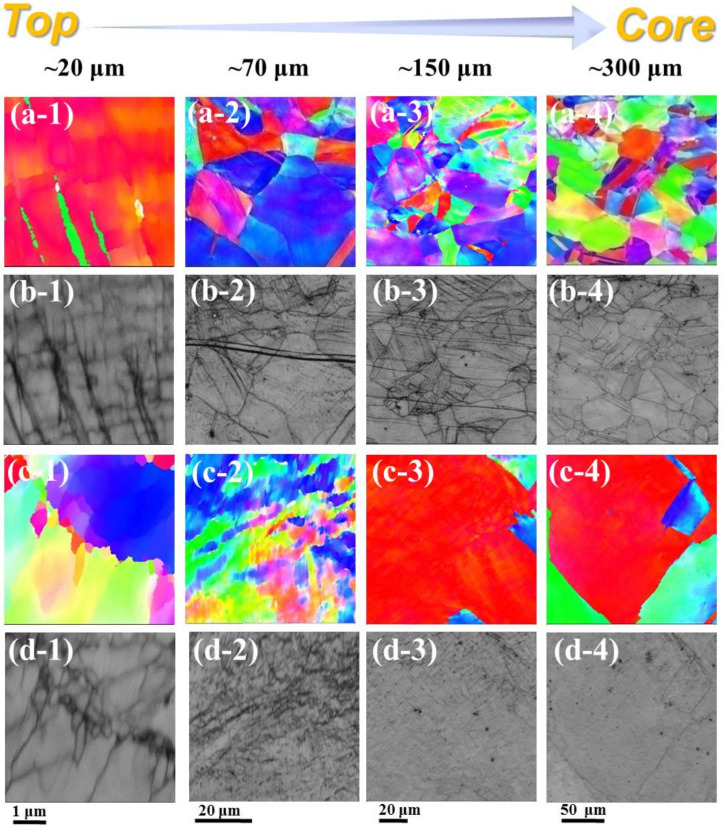
EBSD maps showing gradient microstructures at the depth range between ~20 μm and ~300 μm from the surfaces: (**a-1**–**a-4**) IPF map of RASP-MEA, (**b-1**–**b-4**) channeling contrast map of RASP-MEA, (**c-1**–**c-4**) IPF map of RASP-Ni, and (**d-1**–**d-4**) channeling contrast map of RASP-Ni.

RASP imposed both high strain and high strain rate on the surfaces of the sample materials. As a result, gradient nano-structures formed on the sample surfaces, as shown in Figure 2. The topmost surface of the sample experienced the highest strain and strain rate [43,44], and thus extreme grain refinement is expected at a depth of less than ~20 μm. However, the resolution of EBSD is insufficient for acquiring the actual nano-structure at the topmost surface. Hence, blurry EBSD images were obtained at the depth of ~20 μm, as shown in Figure 2(a-1,b-1,c-1,d-1). In spite of the limited quality of the images, defects in nano-scale are noticeable by the channeling contrast in Figure 2(b-1,d-1). Interestingly, the defects at the surface of the RASP-MEA appeared as straight dark lines under the channeling contrast, indicating that the nano-structures are related to confined dislocation slip and/or deformation twinning [44]. In contrast, the defects at the surface of the RASP-Ni appeared as cell-like structures, indicating that the nano-structures are related to dislocation sub-structures [45].

**Figure 3:**
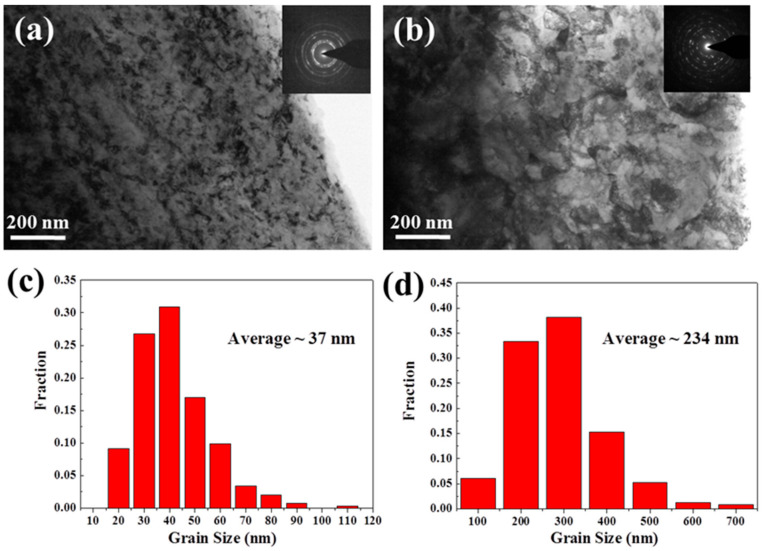
TEM images showing the microstructures at the surfaces of (**a**) the RASP-MEA sample and (**b**) the RASP-Ni sample (SAED patterns are provided as inserts). Charts showing grain size distributions at the surface regions of (**c**) the RASP-MEA sample and (**d**) the RASP-Ni sample.

At the depths of ~70 μm and 150 μm from the surface of the RASP-MEA, coarse grains and planar defects feature in the microstructure, as shown in Figure 2(a-2,a-3,b-2,b-3). This also indicates that grain refinement was only achieved at the depth of a few tens of micrometers, possibly less than 50 μm. In contrast, at the depth of ~70 μm from the surface of RASP-Ni, equiaxed sub-grains feature in the microstructure. Therefore, it can be concluded that under the same shot peening impact energy, the depth of grain refinement is shallower in MEA than in CP-Ni. In RASP-Ni, the coarse grains could be sustained at the depth of 150 μm. However, the moderate color variation within the grain shown in Figure 2(c-3) suggests that dislocation entanglement is pronounced at the grain interior.

**Figure 4:**
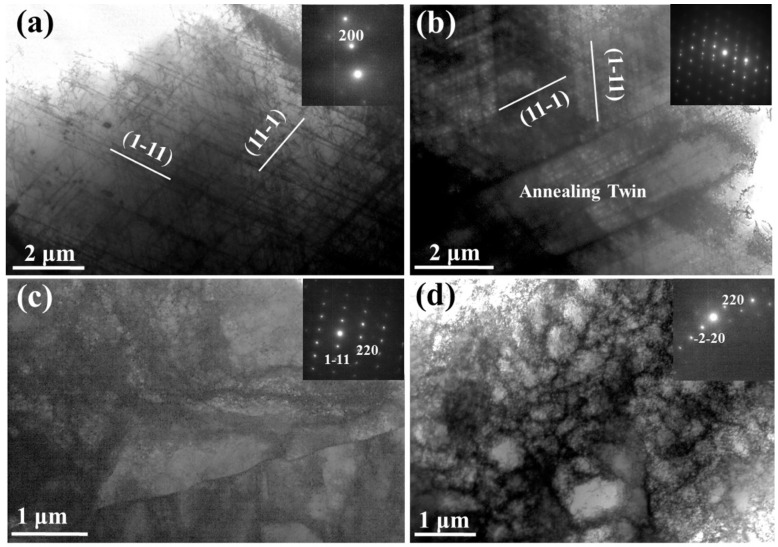
TEM images showing typical microstructures at the depth of ~300 μm from the surfaces of (**a,b**) RASP-MEA and (**c,d**) RASP-Ni (SAED patterns are provided as inserts).

At approximately 300 μm below the surface of the RASP-MEA, the defect densities are lower than those at the 150 μm depth, evidenced by the moderate variation of local misorientation (Figure 2(a-4)) and the low density of defect lines (Figure 2(b-4)). Similarly, the defect density at ~300 μm below the surface of the RASP-Ni is also comparatively low, in spite of the large grain size, as shown in Figure 2(c-4,d-4).

TEM analysis was conducted to reveal the microstructural details that are hardly resolved by EBSD. Figure 3a is a TEM image, and shows the corresponding selected area electron diffraction (SAED) pattern obtained at the surface layer (<20 μm from the surface) of the RASP-MEA. Uniformly distributed nano-grains, shown by the bright-field TEM image, and the diffraction rings shown by the SAED pattern together reveal that extreme grain refinement has been achieved at the surface of the RASP-MEA sample. Statistical analysis based on a series of TEM images produced the grain size distribution chart shown in Figure 3c. The average grain size at the surface is estimated to be 37 nm, which is towards the lower bond of the nano-crystalline regime [46,47,48]. In contrast, the grain refinement at the surface layer of RASP-Ni is not as significant as in RASP-MEA. Figure 3b shows ultrafine grains at the surface layer of RASP-Ni. Many of the ultrafine grains have diffused grain boundaries due to severe lattice distortion [49]. Figure 3d shows that the average grain size is ~230 nm at the surface layer of RASP-Ni. Thus, it can be concluded here that the effectiveness of surface nano-crystallization of FeCoNiMoV MEA under RASP processing is much higher than for single-phase materials with high SFE, such as Ni.

At the depth of ~300 μm from the surface of the RASP-MEA sample, the planar dislocation slip along {111} is the major deformation structure, as shown in Figure 4a. Nano-twins are seldom found at this depth, indicating that the shear stress at the depth of 300 μm is insufficient to activate deformation twinning. Twin structures are found, as evidenced by Figure 4b, but they are just annealing twins preserved from the annealed state. For the purpose of comparison, typical microstructures at the depth of ~300 μm from the surface of RASP-Ni are provided in Figure 4c,d. Dislocation wall (Figure 4c) and cell structures (Figure 4d) are the major deformation structures at this depth. It is well known that planar dislocations, dislocation walls and cells form at the early stages of plastic deformation when the strain is very low [29]. Thus, it is believed that impact energy has been mostly absorbed at the depth of 300 μm for both MEA and Ni.

## 4. Discussion

**Figure 5:**
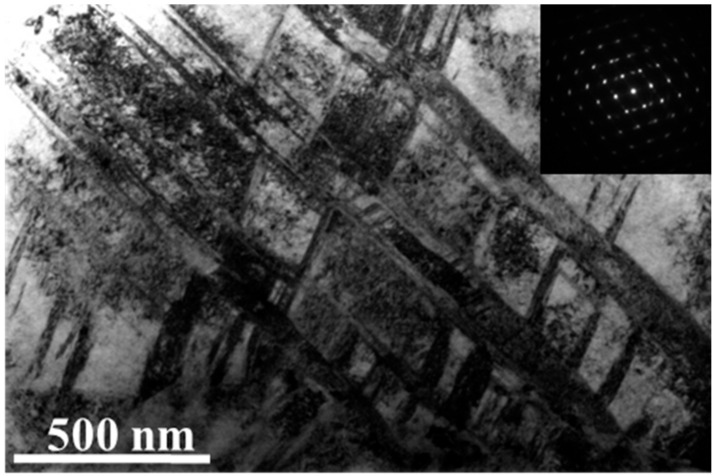
A TEM image showing deformation twins at the depth of 20–40 μm below the surface of RASP-MEA.

Microstructural characterization by both TEM and SEM reveals that planar dislocation slip is the major deformation mechanism when the FeCoNiMoV MEA was deformed by RASP. It is known that the SFE of FeCoNi_2_Mo_0.2_V_0.5_ MEA is ~50 mJ/m^2^ [50], which is comparable to copper. Thus, deformation twinning is also expected when strain and/or strain rate is sufficiently high [51]. Interestingly, deformation twins have only been observed at the depth range of 20 to 40 μm below the surface of RASP-MEA, as shown in Figure 5. This is because the SFE of the FeCoNiMoV MEA is still comparatively high. Hence, very high stress is required to activate deformation twinning. Both the shear stress and shear strain imposed by RASP are very high at the surface of impact, resulting in the quick formation of thin nano-crystalline layers, as shown in Figure 3. Once the nano-crystalline layer is formed, the material’s surface is significantly hardened. The RASP-imposed shear stress decreased drastically when it transmitted through the hard nano-crystalline layer [52]. Thus, only a thin layer of a few tens of nanometers below the nano-crystalline layers experienced a high shear stress, which was just sufficient to activate deformation twinning. As the shear stress decreased further with increasing depth, only dislocation slips and stacking faults could be activated, as shown in Figure 4. Although the deformation twins existed only at the very surface of the RASP-MEA, it is still an important mechanism that facilitates grain refinement at a much faster rate than a grain refinement mechanism via dislocation activities [29]. This concept is also supported by the first-hand experimental results provided here. Ni is a representative high SFE material [29]. Under the same RASP condition, the microstructure of the material’s surface was only refined to the ultrafine-grained regime. This is because high stacking fault energy facilitates cross slip and recovery [29,53,54]. At the very surface of the Ni sample, grain refinement and grain growth are balanced under the shot peening condition. Without any change of the shot peening condition, further grain refinement is impossible, similar to the equilibrium state under high-pressure torsion processing [31,53,55]. Clearly, the ultrafine grained surface layer is less effective in absorbing impact energy than the nanocrystalline layer. As such, the high strain energy is transmitted to a deeper region of 70 μm below the surface of RASP-Ni to cause grain refinement, as shown in Figure 2 (c-2,d-2).

## 5. Conclusions

In summary, the Ni_2_FeCoMo_0.5_V_0.2_ MEA and CP-Ni treated by RASP were characterized by EBSD and TEM. Nano-grains with an average size of 37 nm were obtained on the surface layer of the Ni_2_FeCoMo_0.5_V_0.2_ MEA. Microstructural analysis shows that deformation twinning and dislocation activities are closely involved in the grain refinement mechanism. In contrast, only dislocation activities contributed to the grain refinement of CP-Ni, leading to the ultrafine grained surface layer. RASP exhibited a prominent structure refinement ability for MEA, and successfully produced gradient nano-structured MEA samples.
